# The Effect of Preoperative Intravenous Iron Supplementation on Mortality and Blood Transfusion Requirements in Elderly Patients Undergoing Hip Fracture Surgery: A Prospective Randomized Controlled Trial

**DOI:** 10.3390/jcm14134713

**Published:** 2025-07-03

**Authors:** Taha Kizilkurt, Mustafa Ozkaya, Mert Balli, Mehmet Demirel, Mehmet Asik

**Affiliations:** Department of Orthopaedics and Traumotology, Faculty of Medicine, Istanbul University, Çapa, 34093 Istanbul, Turkey

**Keywords:** ferric carboxymaltose, hip fracture, restrictive transfusion, mortality

## Abstract

**Background/Objectives**: Elderly patients who suffer a hip fracture often have a high risk of complications and mortality, which can be made worse by anemia during and after surgery. Although restrictive transfusion strategies are recommended, the role of preoperative intravenous iron, particularly ferric carboxymaltose (FCM), remains unclear. This study aimed to investigate whether preoperative IV FCM reduces mortality and transfusion requirements in geriatric hip fracture patients managed under a restrictive transfusion strategy. **Methods**: A study was conducted in which 220 patients aged 65 years and over who had undergone surgery for a hip fracture were included. These patients were allocated to receive either a single 1000 mg dose of intravenous FCM approximately 12 h before surgery or no iron supplementation. All the patients were managed with a standardized restrictive transfusion strategy. The primary outcome was all-cause mortality at 6 and 12 months. The secondary outcomes included perioperative transfusion requirement, hemoglobin trends, and length of hospital stay. **Results**: The FCM group demonstrated significantly lower mortality at both 6 months (22.9% vs. 39.0%, *p* = 0.011) and 12 months (28.4% vs. 42.9%, *p* = 0.028) compared to the control group. Multivariate logistic regression identified preoperative FCM administration as one of the independent protective factors for mortality. The FCM group had significantly lower transfusion rates (30.9% vs. 45.5%, *p* = 0.02). No significant difference was observed at the 6-week follow-up in terms of the higher discharge hemoglobin levels seen in the control group. The difference in hospital stay duration did not reach statistical significance. **Conclusions**: Preoperative intravenous FCM administration could reduce both short- and long-term mortality and transfusion needs in geriatric hip fracture patients managed under a restrictive transfusion protocol. These findings support further investigation of high dose IV iron as a component of perioperative blood management in this high-risk population.

## 1. Introduction

Hip fractures are among the most serious injuries that can affect older people, and the number of cases is expected to rise sharply as the global population ages. The number of hip fractures is expected to rise from 1.7 million in 1990 to 6.3 million by 2050 [1]. Allogeneic blood transfusion remains a frequently employed intervention in geriatric patients undergoing surgery for hip fractures, largely due to perioperative blood loss and a high prevalence of pre-existing anemia [2,3]. Nonetheless, allogeneic blood transfusion in geriatric hip fracture patients carries a risk of adverse outcomes, including higher rates of postoperative urinary tract infections [4], prolonged hospital stays [5], and potentially increased long-term mortality [6,7]. Accordingly, in an effort to minimize the need for blood transfusion and mitigate transfusion-related morbidity in this vulnerable patient population, several strategies have been employed under the framework of patient blood management. These strategies comprise preoperative anemia screening and treatment [8]; intraoperative blood-sparing techniques such as tranexamic acid administration [9]; and restrictive transfusion thresholds [10].

Among the pharmacologic blood management approaches, intravenous ferric carboxymaltose (FCM) has been shown to effectively reduce the need for ABT and improve postoperative hemoglobin in various surgical settings, including cardiac [11] and abdominal surgery [12]. It also offers practical advantages, including the ability to administer 1 g in a single 15 min infusion, equivalence to 1000 mg iron sucrose, and a favorable safety and tolerability profile [13,14]. Additionally, FCM may confer additional clinical benefit by correcting iron deficiency anemia, which is a prevalent and clinically significant condition associated with impaired postoperative recovery, delayed rehabilitation, prolonged hospitalization, and increased mortality [15]. Despite its established benefits in transfusion reduction and anemia correction, our literature review identified a notable gap. Although previous studies have explored iron supplementation after hip fracture surgery, evidence on mortality impact remains lacking. Parker et al. [2] found no benefit of oral iron in a randomized trial, while Yoon et al. [16] showed reduced transfusion rates with preoperative IV FCM in a retrospective study, but no mortality difference. Additionally, Cuenca et al. [17] reported reduced transfusion and 30-day mortality with preoperative iron sucrose, though in a non-randomized design and using a different iron formulation. As far as we know, no one has conducted a randomized controlled trial to evaluate whether preoperative IV iron impacts mortality in this vulnerable population.

The main aim of this study was to see if giving FCM before surgery can reduce the elderly patient mortality within a year of having hip surgery. The secondary aim included assessing the effects of FCM on perioperative transfusion requirements, postoperative hemoglobin levels, length of hospital stay, and early postoperative complications. We hypothesized that a single preoperative dose of FCM would be associated with lower postoperative 6-month and 1-year mortality, decreased perioperative transfusion requirements, and improved perioperative outcomes compared to standard care without iron supplementation.

## 2. Patients and Methods

### 2.1. Study Design and Eligibility Criteria

The study was planned as a prospective trial where people were randomly divided into control and intervention groups, and was conducted at a university hospital between October 2023 and May 2024. Review and approval of the study protocol was granted by the Clinical Research Ethics Committee of Istanbul University (Approval No. 1916246, approval date: 10 July 2023), and the Declaration of Helsinki was adhered to in the conducting of all study procedures. Prior to enrolment, all the participants provided written informed consent. The trial was registered at ClinicalTrials.gov (Registry number NCT06080893).

Patients met the inclusion criteria if they were (1) aged 65 years or over and (2) had a hip fracture requiring surgical intervention. The following were considered to be the exclusion criteria: (1) history of iron therapy during hospitalization or iron supplementation in the last three months; (2) receipt of any blood transfusion prior to surgery; (3) presence of a pathological fracture; (4) multiple trauma involving other long bones or anatomical regions; (5) known allergy or documented hypersensitivity to any intravenous iron formulation; and (6) unwillingness to participate in the study.

### 2.2. Randomization and Intervention

Patients were randomly allocated into two groups using sealed, opaque, sequentially numbered envelopes: the FCM group and the control group. Randomization was performed after confirmation of eligibility, and the treating physicians were blinded to group assignment. A single 1000 mg IV dose of FCM was administered to patients approximately 12 h before surgery, via slow infusion over 20–30 min [12]. The control group received no placebo or iron preparation. No patient in either group received tranexamic acid, either intravenously or topically, and no oral iron supplementation was administered during the perioperative period.

### 2.3. Perioperative Transfusion Protocol

All the patients were managed according to a restrictive transfusion strategy. Transfusions were permitted only if the hemoglobin level fell below 8 g/dL or if patients developed clinical signs of anemia, such as chest pain of cardiac origin, congestive heart failure, unexplained tachycardia, or hypotension unresponsive to fluid therapy [18]. The patient received blood one unit at a time, with their clinical status being reassessed before each additional transfusion. Perioperative transfusion requirements—including both intraoperative and postoperative transfusions up to hospital discharge—were recorded, including the number of transfused units and the number of patients receiving transfusions. The term perioperative refers to transfusions administered intraoperatively and postoperatively during the follow-up period. Transfusion status was further categorized as follows: no transfusion, transfusion with 1–2 units, and transfusion with ≥3 units of erythrocyte suspension (ES replacement).

### 2.4. Outcome Measures and Follow-Up Protocol

The primary focus of the study, which looked at hip fracture patients over the age of 65, was mortality rates after hip surgery in the first six months and one year following the procedure. Secondary outcomes included preoperative anemia status, postoperative anemia status, preoperative hemoglobin levels (measured at time of admission), postoperative hemoglobin levels (measured 24 h after surgery), hemoglobin levels at discharge, and length of hospital stay (LOS). Hemoglobin levels were reassessed at the sixth postoperative week in patients who did not receive any additional iron supplementation during the follow-up period.

Mortality follow-up was performed at both 6 and 12 months postoperatively through scheduled outpatient visits or structured telephone interviews. When necessary, mortality status was verified using hospital records and national health registry databases to ensure accuracy. All surviving patients were followed for at least 12 months.

Baseline characteristics—including age, sex, body mass index (BMI), type of fracture, American Society of Anesthesiologists (ASA) classification [19], type of surgical procedure (total hip arthroplasy with direct lateral approach, hemiarthroplasty with posterolateral approach, and intramedullar nail), and comorbidities—were prospectively recorded at the time of admission. A detailed comorbidity assessment was also performed for each patient. Evaluated comorbidities included renal disease, hypertension, cardiovascular disease, diabetes mellitus, thyroid dysfunction, pulmonary disease, neurologic disorders (e.g., previous stroke, transient ischemic attack, or Parkinson’s disease), dementia, and malignancy. In addition to individual comorbidities, the total number of comorbid conditions was recorded and categorized as either 0–1 or ≥2. The Charlson Comorbidity Index (CCI) was also calculated to quantify the overall burden of chronic illness and facilitate comparison between groups [20].

Beyond clinical outcomes, the study also evaluated the safety profile of intravenous FCM administration. All patients in the FCM group were prospectively monitored for adverse events, including perioperative phosphat levels, hypersensitivity reactions, infusion-related complications, or other unexpected side effects. Any such events were documented during hospitalization and throughout the follow-up period.

### 2.5. Statistical Analysis

The performance of statistical analyses was performed via IBM SPSS Statistics for Windows, Version 29.0 (IBM Corp., Armonk, NY, USA). The normality of continuous variables was evaluated through the utilization of the Shapiro–Wilk test and histograms. The mean ± standard deviation (SD) and the median with interquartile range (IQR) were used to express continuous variables. The presentation of categorical variables was in the form of frequencies and percentages (%). The independent samples *t*-test was utilized to compare the normally distributed continuous variables between groups. In order to facilitate the comparison of categorical variables, the Pearson’s chi-squared test or Fisher’s exact test was utilized, as appropriate. Within-group comparisons of repeated hemoglobin measurements across time points were analyzed using repeated measures ANOVA or the Friedman test, depending on the distribution. Post hoc pairwise comparisons were conducted using Bonferroni correction where applicable. A backward stepwise elimination method was used to perform multivariate logistic regression analyses to identify independent predictors of 6-month and 1-year mortality. The presentation of results was as odds ratios (OR) with 95% confidence intervals (CI). The G*Power 3.1.Ink software was used to calculate the sample size, with the effect size reported by Cuenca et al. [17], who observed a 19.3% difference in 30-day mortality between standard care and patients receiving perioperative IV iron sucrose. This was based on a moderate effect size (w = 0.5). It also assumed a significance level of 0.05. And it assumed a power of 95%. The study required a minimum sample size of 220 patients. This was split equally between the two groups.

## 3. Results

### 3.1. Baseline Characteristics and Comorbidity Profile

**Figure 1:**
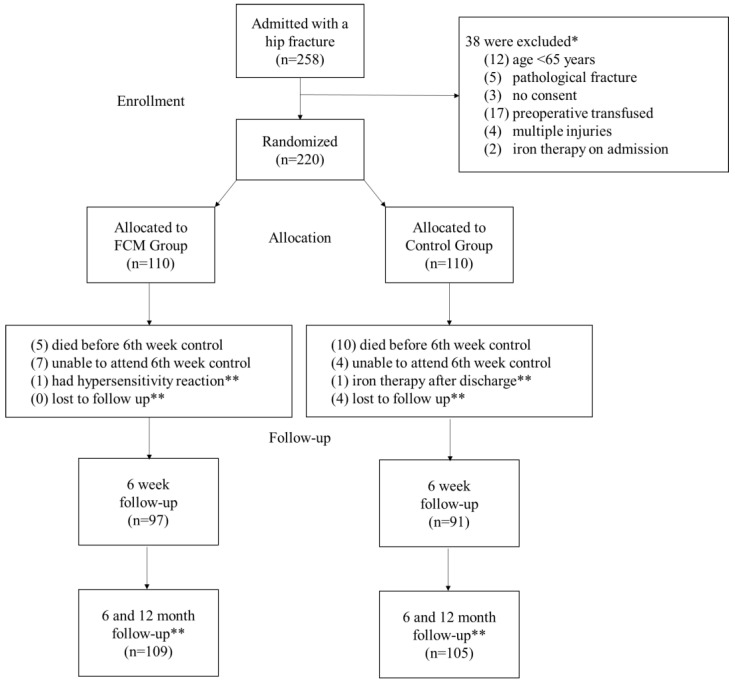
Executive summary of patients included in the study. * The total number of excluded patients is not equal to the sum of all exclusion criteria, as some patients met more than one criterion. ** This refers only to patients who were lost to follow-up at both 6-month and 12-month mortality assessments.

As illustrated in the flowchart (Figure 1), a total of 258 patients presenting with hip fractures were initially screened for eligibility. Following the application of predefined exclusion criteria, 220 patients were enrolled and randomized into the FCM group (n = 110) and the control group (n = 110). Each stage of the study, including group allocation, 6th-week outpatient follow-up, and 6- and 12-month mortality assessments, along with documented reasons for loss to follow-up, is also illustrated by the flowchart, which shows how patients progress through it.

The FCM and control groups were indistinguishable from each other in terms of baseline characteristics and comorbidity profiles, as no statistically significant differences were observed, except for the higher prevalence of dementia in the control group (17% vs. 7%, *p* = 0.016) and the higher phosphate level in the FCM group (mean: 3.51 ± 0.73 mg/dL; median: 3.47 [IQR: 2.93–3.96] vs. mean: 3.27 ± 0.78 mg/dL; median: 3.21 [IQR: 2.82–3.75], *p* = 0.023). Table 1 and Table 2 present the detailed distributions of baseline characteristics and comorbidity variables, respectively.

### 3.2. Primary Outcomes: Mortality Rates

The 6-month mortality rate was 22.9% in the FCM group and 39.0% in the control group (*p* = 0.011). The 1-year mortality rate was 28.4% in the FCM group and 42.9% in the control group (*p* = 0.028). Both time points demonstrated significantly lower mortality in the FCM group (Table 3).

### 3.3. Secondary Outcomes

#### 3.3.1. Anemia Status

In the FCM group, preoperative anemia was observed in 88 patients (81%), while in the control group, it was seen in 86 patients (82%) (*p* = 0.826). Postoperative anemia was present in 107 patients (98%) in the FCM group and 101 patients (96%) in the control group (*p* = 0.382). Anemia at discharge was found in 106 patients (97%) in the FCM group and 99 patients (94%) in the control group (*p* = 0.281). At the 6th postoperative week, anemia was present in 86 patients (89%) in the FCM group (n = 97) and 89 patients (98%) in the control group (n = 91). This was the only time point at which a statistically significant difference was observed between the groups, with a higher proportion of patients being non-anemic in the FCM group (*p* = 0.029) (Table 3).

#### 3.3.2. Hemoglobin Trends over Time

In within-group analyses, the hemoglobin levels differed significantly at different time points in both the FCM and control groups. In the FCM group, the hemoglobin values at preoperative, postoperative, discharge, and 6th-week time points differed significantly (*p* < 0.001). Significant differences between preoperative values and all the subsequent time points were revealed by post hoc pairwise comparisons (all *p* < 0.001), as well as between postoperative and 6th-week levels (*p* = 0.017). A statistically significant difference in hemoglobin levels across time points was observed in the control group (*p* < 0.001). However, when the data was compared in pairs, it was found that there were significant differences only between the preoperative values and those measured at the time of discharge and at the 6th week after surgery (all *p* < 0.05), with no significant differences observed between postoperative and subsequent time points (*p* > 0.05) (Table 3).

Between-group comparisons of hemoglobin levels at each time point revealed no significant differences in the preoperative period (median 10.8 g/dL [IQR: 9.7–12.3] in the FCM group vs. 10.5 g/dL [IQR: 9.2–11.7] in the control group; *p* = 0.427) or immediately postoperatively (9.6 g/dL [IQR: 9.0–10.8] in the FCM group vs. 9.9 g/dL [IQR: 9.0–10.6] in the control group; *p* = 0.159). At discharge, the hemoglobin levels were significantly higher in the control group (mean: 10.29 ± 1.02 g/dL; median: 10.2 [IQR: 9.6–11.4]) compared to the FCM group (mean: 9.78 ± 1.03 g/dL; median: 9.7 [IQR: 7.3–12.4]) (*p* < 0.001). At the 6th postoperative week, no significant difference was observed between the FCM group [10.4 g/dL [IQR: 9.4–11.0]) and the control group (10.3 g/dL [IQR: 9.9–10.9]) (*p* = 0.242) (Table 3).

#### 3.3.3. Perioperative Transfusion Characteristics

A significantly lower rate of ES transfusion was observed in the FCM group (30%, n = 34) compared to the control group (46%, n = 48) (*p* = 0.013). Among those who received transfusions, 1–2 units were administered to 97.1% (n = 33) in the FCM group and 93.8% (n = 45) in the control group. Transfusion of ≥3 units was rare, observed in 2.9% (n = 1) and 6.2% (n = 3) of patients in the FCM and control groups, respectively (Table 3).

#### 3.3.4. Length of Hospitalization

The median length of stay in hospital for those in the control group was 11 days (range: 7–14 days), while for those in the FCM group it was 10 days (range: 6–10 days). The difference in the duration of hospitalization between the two groups was not statistically significant (*p* = 0.250), but the FCM group had a shorter duration of hospitalization. (Table 3).

#### 3.3.5. Adverse Events Related to FCM Administration

No serious adverse events were reported in the FCM group. However, one patient developed a self-limiting mild hypersensitivity reaction, which did not require advanced medical intervention and included non-severe symptoms such as localized rash, itching, mild nasal and ocular symptoms, without systemic or life-threatening involvement, and was subsequently excluded from the study. Considering hypophosphatemia as one of the potential adverse effects of FCM, no statistically significant differences were observed between the groups regarding changes in phosphate levels (mean: 0.10 ± 0.99 mg/dL; median: 0.12 [IQR: 0.71–(−0.58)] vs. mean: 0.13 ± 0.87 mg/dL; median: 0.19 [IQR: 0.69–(−0.48)], *p* = 0.776) or hypophosphatemia status (27 patients; 25% vs. 38 patients; 36%, *p* = 0.069) at discharge.

### 3.4. Multivariate Analysis of Mortality Outcomes

#### 3.4.1. 6-Month Mortality

In the multivariate logistic regression model, preoperative IV FCM administration (OR: 0.330, *p* = 0.003), age (per year increase) (OR: 1.062, *p* = 0.006), female gender (OR: 0.424, *p* = 0.038), CCI score (per point) (OR: 1.398, *p* = 0.014), ASA class (high vs. low) (OR: 2.309, *p* = 0.035), fracture type (femoral neck vs. intertrochanteric) (OR: 0.388, *p* = 0.017), hypertension (present vs. absent) (OR: 6.446, *p* = 0.002), and neurologic disorders (present vs. absent) (OR: 1.292, *p* = 0.037) were independently associated with 6-month mortality (Table 4).

#### 3.4.2. 1-Year Mortality

For 1-year mortality, the final model identified preoperative IV FCM administration (OR: 0.449, *p* = 0.021), age (OR: 1.059, *p* = 0.003), female gender (OR: 0.445, *p* = 0.015), CCI score (OR: 1.248, *p* = 0.019), ASA class (OR: 2.168, *p* = 0.062), hypertension (OR: 3.583, *p* = 0.001), and neurologic disorders (OR: 3.266, *p* = 0.018) as independent predictors (Table 5).

Although there was a statistically significant difference in the prevalence of dementia between the two groups, dementia was not identified as an independent risk factor for 6-month and 1-year mortality in the multivariable logistic regression analyses. This finding suggests that the effect of dementia on mortality may be mediated or confounded by other factors such as age, gender, ASA class, and comorbidities.

## 4. Discussion

Elderly patients who suffer a hip fracture are prone to significant morbidity and mortality, especially when anemia and high transfusion rates are present during the operation. Despite advances in surgical techniques and perioperative care, one-year mortality following hip fracture surgery remains high, as consistently reported in the literature [21,22,23]. Intravenous iron supplementation, particularly with FCM, has been proposed as a promising strategy to optimize preoperative hemoglobin levels and reduce transfusion requirements. While studies in cardiac and abdominal surgeries [11,12] have demonstrated favorable effects on hemoglobin restoration and transfusion reduction, evidence from prospective randomized trials specifically focusing on patients undergoing hip fracture surgery remains limited. Therefore, in this randomized controlled trial, we hypothesized that a single preoperative dose of FCM would reduce 6-month and 1-year postoperative mortality and decrease perioperative transfusion requirements, while also improving short-term clinical outcomes. The results of the study confirmed this hypothesis, demonstrating a significant reduction in 6- and 12-month postoperative mortality, along with lower perioperative transfusion rates in the FCM group compared to the control group.

Previous studies investigating iron supplementation following hip fracture surgery have offered limited insight into clinically meaningful outcomes such as postoperative recovery and mortality [2,16,17]. Most available data are derived from retrospective cohorts, non-randomized comparisons, or studies focusing on oral or alternative intravenous iron formulations. Parker (2010) [2] conducted a randomized trial comparing postoperative oral iron supplementation (200 mg ferrous sulfate twice daily for 28 days, n = 150) with no iron therapy (n = 150) in patients with anemia after hip fracture surgery. The study found no improvement in hemoglobin recovery, transfusion needs, or clinical outcomes, with both groups demonstrating a 1-year mortality rate of 19.3% (29 patients per group). Cuenca (2005) [17] reported encouraging findings from a prospective, non-randomized study comparing patients who received preoperative intravenous iron sucrose (n = 20) with a control group without iron therapy (n = 57) undergoing hemiarthroplasty for displaced subcapital hip fractures. Lower transfusion indices and a significant reduction in 30-day mortality (0% vs. 19.3%, *p* = 0.034) were demonstrated by the iron group, although the study’s limitations were due to the small sample size, lack of randomization, and use of a less potent iron formulation [17]. Yoon et al. (2019) [16] conducted a retrospective cohort study comparing a liberal transfusion strategy (n = 775) with a restrictive protocol incorporating preoperative intravenous iron sucrose (200 mg) (n = 859) in patients over the age of 65 undergoing hip fracture surgery. While the restrictive group showed reduced transfusion rates (48.2% vs. 65.3%, *p* < 0.001), shorter hospital stays (21.5 ± 36.8 vs. 28.8 ± 29.9 days, *p* < 0.001), and higher hemoglobin levels at 6 weeks, there was no significant difference in mortality at 30, 60, or 90 days. The retrospective design and lack of randomization may have limited the study’s ability to detect a survival benefit. In contrast to these studies, the effect of preoperative FCM on both postoperative recovery and 6- and 12-month mortality in geriatric patients undergoing hip fracture surgery has been evaluated in our trial. This is the first prospective, randomized, controlled study to do so. By employing a standardized, high-dose, single-infusion IV iron protocol (1000 mg FCM administered approximately 12 h preoperatively), our study overcomes key methodological limitations of earlier investigations. These design strengths allow for a more definitive assessment of clinical benefit and represent a significant contribution to the evolving evidence base on patient blood management strategies in this vulnerable population. In addition, the effects of preoperative intravenous FCM—either on its own or in combination with erythropoietin—in geriatric anemic patients with hip fractures were evaluated in another study by Bernabeu-Wittel et al. [24]. A significant reduction in transfusion requirements or mortality was not shown in the randomized controlled trial, despite improved hemoglobin recovery and resolution of anemia at 60 days. The discrepancy with our findings may be explained by differences in transfusion thresholds and the co-administration of erythropoietin.

Given that mortality was the primary outcome of this study, the significant reduction observed at both 6 and 12 months in the FCM group represents a clinically and statistically meaningful finding. Importantly, multivariate logistic regression analysis confirmed that preoperative IV iron administration was an independent protective factor for mortality at both time points, even after adjusting for age, comorbidity burden, ASA class, and other known prognostic variables. These findings suggest that the observed survival benefit is not merely a reflection of baseline differences but is likely attributable to the intervention itself. While the mechanisms remain speculative, early correction of functional iron deficiency may enhance physiologic resilience, reduce transfusion exposure, and mitigate downstream complications in frail elderly patients undergoing surgery [12,25,26]. Compared to oral iron, which is frequently associated with gastrointestinal intolerance [2], and iron sucrose, which requires multiple low-dose administrations [27], FCM offers the advantage of delivering a full replacement dose in a single short infusion with favorable tolerability [26]. Although serious adverse reactions to FCM are rare, systemic hypersensitivity remains a known risk [28], as observed in one patient in our study. Another adverse event associated with FCM use is hypophosphatemia, which can be severe and prolonged in some patients, potentially leading to osteomalacia and fractures [29,30]. To explore this safety concern in our cohort, we analyzed serum phosphate levels at the preoperative and discharge time points. Although no significant changes were observed in either group and no clinically relevant cases of hypophosphatemia were detected, it should be noted that hypophosphatemia following FCM administration typically develops 14–21 days post-infusion, and thus, measuring phosphate levels at discharge (median: 10 days) may have resulted in underdetection of delayed-onset cases. Furthermore, since preoperative phosphate levels were already higher in the FCM group, the significant difference observed at discharge was expected and likely reflects baseline variation rather than a treatment effect.

Beyond mortality, several secondary outcomes further support the potential benefit of preoperative FCM administration. The FCM group demonstrated significantly lower rates of perioperative transfusion compared to controls, findings that are consistent with previous studies highlighting the transfusion-sparing effects of intravenous iron therapy [12,16,25]. The significantly higher hemoglobin levels observed at discharge in the control group, compared to the FCM group, may primarily reflect the greater frequency of blood transfusions in this group. Interestingly, hemoglobin values at that point showed a non-significant trend favoring the FCM group, suggesting a possible delayed hematologic benefit. Although a shorter median length of hospital stay was observed in the FCM group, the difference did not reach statistical significance, possibly due to variability in discharge protocols and comorbidity profiles. Nevertheless, the overall pattern of secondary outcomes is consistent with the hypothesis that timely correction of iron deficiency may facilitate early postoperative recovery in geriatric patients undergoing hip fracture surgery.

This study has certain limitations that warrant consideration. A potential limitation of the study is the absence of blinding among outcome assessors. However, as the primary outcomes—mortality and transfusion requirement—were objectively defined and extracted from institutional databases, the risk of detection bias is likely minimal and unlikely to have meaningfully influenced the results. While the sample size was sufficient to detect differences in key clinical outcomes, it may not have been powered to evaluate infrequent adverse events or more nuanced postoperative parameters such as functional status or quality of life. Additionally, the follow-up protocol primarily focused on mortality and hemoglobin dynamics rather than long-term rehabilitation metrics. Despite these limitations, this trial possesses several notable strengths. To our knowledge, it is the first prospective, randomized controlled study to examine the effect of preoperative FCM on mortality and perioperative outcomes in geriatric patients undergoing hip fracture surgery. The use of a standardized transfusion strategy, rigorous randomization, and multivariate adjustment for known prognostic variables enhances the internal validity of the results. Moreover, the focus on clinically meaningful endpoints strengthens its relevance to real-world perioperative care. Importantly, our study includes the largest patient cohort to date examining the optimization of a guideline-based restrictive transfusion strategy supported by preoperative IV FCM administration in geriatric patients with hip fractures.

## 5. Conclusions

Preoperative administration of intravenous FCM in geriatric patients undergoing hip fracture surgery could reduce both 6-month and 1-year mortality, as well as the need for perioperative blood transfusion. These findings suggest that preoperative IV FCM may contribute to improved survival outcomes in this high-risk population, even in the absence of significant short-term hemoglobin improvement. Further clinical trials are warranted to explore the integration of preoperative high-dose IV iron into restrictive transfusion protocols and to investigate whether its combination with erythropoiesis-stimulating agents could further enhance outcomes in geriatric hip fracture patients.

## Figures and Tables

**Table 1:** Baseline demographic and clinical characteristics of the patients in the FCM and control groups.

Variables		FCM Group	Control Group	*p* Values
**Number of patients**	n	109	105	
**Age (years)**	Mean ± SD Median (IQR)	78.19 ± 10.79 80.0 (71.0–87.0)	78.91 ± 9.20 79.0 (74.0–88.0)	0.98
**Gender**	(n, %)			
Female		65 (60%)	68 (65%)	0.44
Male		44 (40%)	37 (35%)	
**BMI** (kg/m^2^)		29.5 (±2.3) 29 (28.0–31.0)	28.8 (±3.1) 28.5 (26.5–31.0)	0.19
**ASA grade**	(n, %)			
I		8	6	0.290
II		45	43	
III		50	51	
IV		6	5	
**Fracture location**	(n, %)			
Femoral neck fracture		39 (36%)	38 (36%)	0.53
Intertrochanteric fracture		70 (64%)	67 (64%)	
**Surgery**	(n, %)			
Hemiarthroplasty		35 (32.1%)	31 (29.5%)	0.658
Total hip arthroplasty		6 (5.5%)	9 (8.6%)	
Intramedullary nail		68 (62.4%)	65 (61.9%)	
**Preoperative hemoglobin level** (g/dL)	Mean ± SD Median (IQR)	10.78 ± 1.74 10.85 (9.6–12.0)	10.47 ± 1.96 10.55 (9.2–11.7)	0.955
**Preoperative anemia**	(n, %)	88 (81%)	86 (82%)	0.826
**Preoperative phosphate level** (mg/dL)	Mean ± SD Median (IQR)	3.51 ± 0.73 3.47 (2.93–3.96)	3.27 ± 0.78 3.21 (2.82–3.75)	**0.023**
**Preoperative hypophosphatemia**	(n, %)	19 (17%)	26 (25%)	0.188

Abbreviations: SD, standard deviation; IQR, interquartile range; BMI, body mass index; ASA, American Society of Anesthesiologists. Continuous variables are presented as mean ± standard deviation and median (interquartile range), whilst categorical variables are given as number (percentage). The Mann–Whitney U test was used for the comparison of continuous variables due to non-normal distribution. Pearson’s chi-squared or Fisher’s exact test was used to analyze categorical variables. The significance of a *p*-value was indicated by a value of less than 0.05. Anemia is defined as a hemoglobin level of less than 13 g/dL in males and less than 12 g/dL in females. Hypophosphatemia definition: phosphate < 2.8 mg/dL.

**Table 2:** Comparison of comorbidities between the FCM and control groups.

Comorbidities	FCM Group (n = 109)	Control Group (n = 105)	*p* Values
**Number of comorbidities**			
0–1	32 (29%)	31 (30%)	0.979
≥2	77 (71%)	74 (70%)	
**Renal dysfunction**	11 (10%)	12 (11%)	0.752
**Hypertension**	78 (72%)	71 (68%)	0.531
**Cardiac disease ^a^**	36 (33%)	32 (31%)	0.689
**Diabetes Mellitus**	33 (30%)	35 (33%)	0.631
**Thyroid dysfunction**	12 (11%)	13 (12%)	0.910
**COPD**	20 (19%)	13 (12%)	0.149
**Neurological disorders ^b^**	17 (16%)	13 (12%)	0.498
**Dementia**	7 (7%)	19 (17%)	***0.016***
**Malignancy**	10 (9%)	6 (6%)	0.336
**CCI,** Mean ± SD Median (IQR)	4.81 ± 1.68 5 (2–4)	4.58 ± 1.52 4 (1–2)	0.326

Abbreviations: COPD, chronic obstructive pulmonary disease; CCI, Charlson Comorbidity Index; IQR, interquartile range. Categorical data are presented as numbers (percentages). Categorical variables were analyzed using Pearson’s chi-square or Fisher’s exact test. The Mann–Whitney U test was used for the comparison of CCI. The significance of a *p*-value was indicated by a value of less than 0.05. Bold and italic values indicate statistically significant differences. ^a^ A history of ischaemic heart disease, myocardial injury, congestive heart failure, and atrial fibrillation was indicative of cardiac disease; ^b^ A history of stroke, transient ischaemic attacks, Parkinson’s disease, or neuromuscular disease was indicative of neurological disorders.

**Table 3:** Comparison of primary and secondary outcomes between groups.

Variables		FCM Group (n = 109)	Control Group (n = 105)	*p* Values
**Primary Outcomes**				
**6-month mortality rate**	n (%)	25 (23%)	41 (39%)	***0.011***
**1-year mortality rate**		31 (28%)	45 (43%)	***0.028***
**Secondary Outcomes**				
**Anemia rates**				
**Preoperative**	n (%)	88 (81%)	86 (82%)	0.826
**Postoperative**		107 (98%)	101 (96%)	0.382
**At discharge**		106 (97%)	99 (94%)	0.281
**At 6th week**		86 (89%)	89 (98%)	***0.029***
**Hemoglobin levels (g/dL)**				
**Preoperative**	Mean ± SD Median (IQR)	10.76 ± 1.74 10.8 [9.7–12.3]	10.46 ± 1.96 10.5 [9.2–11.7]	0.427
**Postoperative**		9.70 ± 1.35 9.6 [9.0–10.8]	9.91 ± 1.20 9.9 [9.0–10.6]	0.159
**At discharge**		9.78 ± 1.03 9.7 [7.3–12.4]	10.29 ± 1.01 10.2 [9.6–11.4]	***0.001***
**At 6th week**		11.57 ± 1.16 10.4 [9.4–11.0]	10.30 ± 0.91 10.3 [9.9–10.9]	0.242
**Perioperative transfusion characteristics**				
**Transfused patients** (ES replacement)	n (%)	34 (30%)	48 (46%)	***0.013***
1–2 units		33 (97%)	45 (94%)	
≥3 units		1 (3%)	3 (6%)	
**Length of hospitalization (days)**	Mean ± SD Median (IQR)	12.19 ± 9.58 10 [6–10]	15.14 ± 12.7 11 [7–14]	0.250
**Phosphate level at discharge (mg/dL)**		3.42 ± 1 3.41 [2.79–4.05]	3.14 ± 0.79 3.06 [2.6–3.64]	**0.029**
**Change in phosphate levels at discharge (mg/dL)**		0.10 ± 0.99 0.12 [0.71–(−0.58)]	0.13 ± 0.87 0.19 [0.69–(−0.48)]	0.776
**Hypophosphatemia at discharge**	n (%)	27 (25%)	38 (36%)	0.069

The significance of a *p*-value was indicated by a value of less than 0.05. Bold and italic values indicate statistically significant differences. Anemia is defined as a hemoglobin level of less than 13 g/dL in males and less than 12 g/dL in females.

**Table 4:** Multivariate logistic regression analysis identifying independent predictors of 6-month mortality in geriatric hip fracture patients.

Variable Included in Step 12	OR (Exp(B))	95% CI	*p* Values
**Preoperative IV FCM administration** *(Received* vs. *Not received)*	0.330	0.158–0.690	***0.003***
**Age** *(per year increase)*	1.062	1.017–1.109	***0.006***
**Gender** *(female* vs. *male)*	0.424	0.188–0.956	***0.038***
**Fracture type** *(femoral neck* vs. *intertrochanteric)*	0.388	0.179–0.843	***0.017***
**CCI score** *(per point)*	1.398	1.070–1.827	***0.014***
**ASA class** *(high* vs. *low)*	2.309	1.063–5.016	***0.035***
**Hypertension** *(Present* vs. *Absent)*	6.446	2.004–20.735	***0.002***
**Neurologic disorders** *(Present* vs. *Absent)*	1.292	1.092–1.926	***0.037***
**Surgery:** *THA* vs. *Hemiarthroplasty*	0.203	0.023–1.785	0.150
**Surgery:** *IMN* vs. *Hemiarthroplasty*	1.257	0.448–3.526	0.659
**ES transfusion** *(Received* vs. *Not received)*	2.074	0.988–4.355	0.054

Abbreviations: ASA, American Society of Anesthesiologists physical status classification; CCI, Charlson Comorbidity Index; THA, Total Hip Arthroplasty; IMN, Intramedullary Nailing; ES, erythrocyte suspension; FCM, ferric carboxymaltose. Note: Odds ratios (OR) and 95% confidence intervals (CI) were derived from backward stepwise binary logistic regression analysis. The significance of a *p*-value was indicated by a value of less than 0.05. Bold and italic values indicate statistically significant differences. The initial model included the following variables: age, gender, preoperative hemoglobin level, CCI score, ASA class, fracture type (femoral neck vs. intertrochanteric), surgery type (THA and IMN vs. hemiarthroplasty), preoperative IV FCM administration, ES transfusion, renal comorbidity, hypertension, diabetes mellitus, cardiac disease, COPD, dementia, neurological disorders, thyroid dysfunction, and malignancy.

**Table 5:** Multivariate logistic regression analysis identifying independent predictors of 1-year mortality in geriatric hip fracture patients.

Variable Included in Step 13	OR (Exp(B))	95% CI	*p* Values
**Preoperative IV FCM administration** *(Received* vs. *Not received)*	0.449	0.227–0.887	***0.021***
**Age** *(per year increase)*	1.059	1.020–1.100	***0.003***
**Gender** *(female* vs. *male)*	0.445	0.233–0.849	***0.015***
**CCI score** *(per point)*	1.248	1.036–1.504	***0.019***
**ASA class** *(high* vs. *low)*	2.309	1.063–5.016	***0.035***
**Hypertension** *(Present* vs. *Absent)*	3.583	1.676–7.656	***0.001***
**Neurologic disorders** *(Present* vs. *Absent)*	3.266	1.230–8.673	***0.018***
**ES transfusion** *(Received* vs. *Not received)*	1.995	0.938–4.243	0.070

Abbreviations: ASA, American Society of Anesthesiologists physical status classification; CCI, Charlson Comorbidity Index; ES, erythrocyte suspension; FCM, ferric carboxymaltose. Note: Odds ratios (OR) and 95% confidence intervals (CI) were derived from backward stepwise binary logistic regression analysis. The significance of a *p*-value was indicated by a value of less than 0.05. Bold and italic values indicate statistically significant differences. The initial model included the following variables: age, gender, preoperative hemoglobin level, CCI score, ASA class, fracture type (femoral neck vs. intertrochanteric), surgery type (THA and IMN vs. hemiarthroplasty), preoperative IV FCM administration, ES transfusion, renal comorbidity, hypertension, diabetes mellitus, cardiac disease, COPD, dementia, neurological disorders, thyroid dysfunction, and malignancy.

## Data Availability

The original contributions presented in this study are included in the article. Further inquiries can be directed to the corresponding author.

## Abbreviations

The following abbreviations are used in this manuscript:

ASA	American Society of Anesthesiologists physical status classification;
BMI	Body Mass Index;
CCI	Charlson Comorbidity Index;
COPD	Chronic Obstructive Pulmonary Disease;
ES	Erythrocyte Suspension;
FCM	Ferric Carboxymaltose;
IMN	Intramedullary Nailing;
THA	Total Hip Arthroplasty.
